# Reciprocal MicroRNA Expression in Mesocortical Circuit and Its Interplay with Serotonin Transporter Define Resilient Rats in the Chronic Mild Stress

**DOI:** 10.1007/s12035-016-0107-9

**Published:** 2016-09-22

**Authors:** Dariusz Zurawek, Maciej Kusmider, Agata Faron-Gorecka, Piotr Gruca, Paulina Pabian, Joanna Solich, Magdalena Kolasa, Mariusz Papp, Marta Dziedzicka-Wasylewska

**Affiliations:** Department of Pharmacology, Institute of Pharmacology, Polish Academy of Sciences, Smetna Street 12, 31-343 Krakow, Poland

**Keywords:** MicroRNA, Stress, Depression, Resilient, Chronic mild stress, Serotonin transporter

## Abstract

Prolonged stress perturbs physiological balance of a subject and thus can lead to depression. Nevertheless, some individuals are more resilient to stress than the others. Defining molecular factors underlying resilience to stress may contribute to the development of a new antidepressant strategy based on the restoration of resilient phenotype in depressed subjects. We used chronic mild stress (CMS) paradigm—well-characterized animal model of depression which caused in rats behavioral deficits (anhedonia) manifested by decreased consumption of sucrose solution. CMS also generated a proportion of resilient rats which did not alter sucrose consumption despite being stressed. Recently, regulation of a gene expression associated with microRNA (miRNA) is considered as an important factor modulating biochemical response to stress. Based on our previous work and literature survey, we investigated changes in the expression level of seven miRNAs (i.e., miR-18a-5p, miR-34a-5p, miR-135a-5p, miR-195-5p, miR-320-3p, miR-674-3p, miR-872-5p) in mesocortical circuit—crucially involved in stress response in order to find differences between susceptible and resilient phenotype. Bioinformatic analysis showed that all miRNAs of interest potentially target serotonin transporter (SERT). Chronic stress caused global increase in the expression of the abovementioned miRNAs in ventral tegmental area (VTA) of stressed rats followed by parallel decrease in miRNA expression in prefrontal cortex (PCx). This effect was more profound in resilient than anhedonic animals. Moreover, we observed decreased level of SERT in VTA of resilient rats. Our findings show that mesocortical circuit is involved in the response to stress and this phenomenon is more efficient in resilient animals.

## Introduction

Stress is defined as conditions which perturb the psychological and physiological balance of an individual. Exposure to prolonged periods of stress can lead to mental illnesses such as depression or anxiety. It has been established that some individuals are more resilient to stress than others, and these variations in response to stress concern not only humans but also mammals. It has been shown that susceptibility to stress as well as resilience depends on a combination of genetic and non-genetic factors which interact in complex ways [1]. However, molecular mechanisms underlying stress response are not fully understood. Recently, epigenetic regulation of a gene expression associated with action of small RNA transcripts, named microRNA (miRNA), has come to be considered an important factor contributing to biochemical response to stress in dynamic, complex, and pleiotropic manner [2]. miRNAs, widely expressed in eukaryotes, are small (17–24 nucleotides), non-coding RNA molecules which play important role in posttranscriptional regulation of a diversity of genes including brain-specific targets [3]. miRNAs mainly bind to the three prime untranslated region (3′-UTR) of targeted messenger RNA (mRNA) and, by recruitment of a protein complex named RNA-induced silencing complex (RISC), repress mRNA translation. [4, 5]. It is worth noting that animal miRNAs act throughout partial complementarity. A short specific sequence consisting of seven nucleotides (from positions 2 to 8) in 5′ end of miRNA molecule (named seed region) defines miRNA specificity to targeted mRNA [5]. Therefore, one miRNA may regulate translation of more than one protein, and one mRNA may be targeted by many different miRNAs. Their pleiotropic character of action as well as their ubiquity makes miRNAs perfectly suited regulators of homeostatic state during stress [6].

Recent studies have revealed that alterations of various miRNAs in the brain as well as in peripheral tissues, such as blood, may be associated with stress response [7, 8], schizophrenia, depression [9], and/or treatment with antidepressant drugs [10, 11]. Moreover, altered expressions of miR-18a, miR-124, miR-343-3p, miR-16, miR-141, miR-182, and miR-200a in the brain tissue of suicidal patients have been reported [6]. More recently, it has been reported that depressed patients exhibit disturbed levels of miR-320 and miR-451 in blood as compared to healthy controls [12]. Although much is known on the role of miRNAs in pathology of mental illness, knowledge about the role of miRNA molecules in the stress resilience phenomenon is still rising. Issler et al. have shown that miR-135a has been associated with stress resilience and antidepressant treatment. The authors even postulate that miR-135a may act as an endogenous antidepressant [13].

To further explore the issue concerning involvement of miRNA action in response to stress, we used chronic mild stress (CMS) paradigm, which is a well-considered animal model of depression with good face, predictive and construct validity [14]. Chronic exposure of rats to mild unpredictable stressors causes behavioral deficits (anhedonia) which can be reversed by chronic treatment with antidepressant drugs [15–17]. Moreover, in CMS model, there is a proportion of animals which demonstrates resilience to the stress regimen which manifests as unaltered consumption of palatable sucrose solution under stressful conditions [18, 19]. As such, the CMS model seems to mimic natural variety in behavioral response to stress observed among people since not all subjects develop depression despite experiencing prolonged stress. Previously, we have shown that stress-resilient animals exhibited elevated level of miR-16 in serum together with alterations in the expression level of miR-16 in the ventral tegmental area (VTA) and prefrontal cortex (PCx) during different time points of the stress procedure [20]. These results were interesting, as mesocortical and mesolimbic pathways are strongly involved in stress response [21–23]. Moreover, it has been shown that miR-16 negatively regulates the level of serotonin transporter (SERT) in the brain [11]. Thus, it can be postulated that biochemical response to stress, observed especially among resilient subjects, may be regulated by miRNA molecules. To test this hypothesis, in our present study, we decided to look closer at the potential interplay between behavioral stress responses and changes in the expression level of a set of different miRNAs in mesocortical circuit among susceptible and resilient animals and whether these changes may accompany any common biochemical marker associated with pathophysiology of depression. Based on our previous study [20] and literature survey, we chose a set of seven different miRNAs (i.e., miR-18a-5p, miR-34a-5p, miR-135a-5p, miR-195-5p, miR-320-3p, miR-674-3p, miR-872-5p) which are associated with stress response and functioning of the central nervous system.

## Materials and Methods

### Chronic Mild Stress Procedure

Training period as well as CMS procedure was performed according to the method described in detail by Zurawek et al. [24]. In brief, male Wistar Han rats (Charles River, Germany) were first trained to consume a palatable 1 % sucrose solution for six consecutive weeks. During training time, sucrose solution was presented to the rats (twice per week) following overnight food and water deprivation. Sucrose consumption was measured after each 1-h test by reweighing the preweighed bottles containing the sucrose solution. Next, rats (*n* = 220) were randomly divided into the control (*n* = 110) and stressed (*n* = 110) groups. Control animals were housed undisturbed in a separate room with water and food ad libitum except deprivation time before the sucrose test. The stressed group of animals was subjected to the weekly stress regimen for two consecutive weeks. Each weekly stress regimen consisted of two periods of food deprivation, 45° cage tilt, intermittent illumination (lights on and off every 2 h), and low-intensity stroboscopic illumination (150 flashes/min) and one period of water deprivation, one soiled cage (250 ml of water in the sawdust bedding), and paired housing. All stressors lasted for 10–14 h. Sucrose intake was monitored in all groups of rats once per week. Distinctions between anhedonic (*n* = 78) and resilient animals (*n* = 32) were made based on the observation of behavior associated with sucrose consumption of each animal. Particular stressed rat was defined as anhedonic when during stress procedure, sucrose consumption level decreased by more than 20 % as compared to its baseline consumption level (before the start of the CMS) and as compared to consumption observed in non-stressed control group of animals. Resilient rat was defined when its sucrose consumption did not decrease (or even sometimes increased) as compared to its baseline sucrose consumption level (and to consumption in control group of rats) despite the fact that this animal was under stress influence. Then, differences in the sucrose consumption levels by the rats after CMS procedure were retrospectively analyzed by using two-way ANOVA relative to the median split of the baseline sucrose consumption as well as to the control group of animals. For our further biochemical experiments, we randomly chose 14–16 animals from each group after 2 weeks of CMS. The remaining animals were used for another experiment and were not included in this study. All of the behavioral experiments were performed in accordance with the guidelines of the ethical use of animals set by the European Community Council Directive and were approved by the Local Bioethics Commission.

### Serum and Brain Tissue Collection

Of tail vein blood, 0.5 ml was collected from each rat to the Eppendorf tubes at the baseline (i.e., before the start of the CMS paradigm) and after 1 week of CMS. At the end of CMS procedure, trunk blood was collected. Blood samples were kept at room temperature for 30 min to clot and then centrifuged for 20 min at 4 °C at 1500*×g*. After centrifugation, serum was collected to new Eppendorf tubes. Potential occurrence of hemolysis was checked in all serum samples by using NanoDrop spectrophotometer (Thermo Scientific, MA, USA) at 414-nm wavelength absorbance which corresponds to hemoglobin presence. Non-hemolyzed serum samples were frozen and stored at −80 °C until further analysis.

**Fig. 1:**
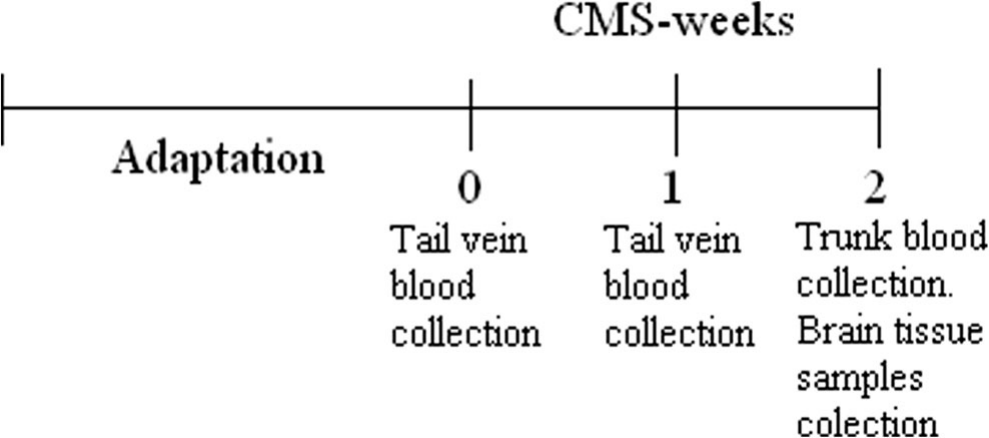
Schematic representation of behavioral experiment and time points in which blood and brain tissue samples were collected

Rats were sacrificed by decapitation, and brains were rapidly removed from the skull. Next, for autoradiographic and in situ hybridization studies, whole rat brains were frozen by using a heptane–dry ice mixture. Then, brains were cut according to The Rat Brain in Stereotaxic Coordinates Atlas (Paxinos and Watson 1998) into 12-μm-thick coronal brain sections through prefrontal cortex (bregma, 3.70 to 1.70 mm), dorsal raphe nucleus (bregma, −7.30 to 8.8.00 mm), and ventral tegmental area (bregma, −5.80 to −6.30 mm) by using a Jung CM 3000 cryostat microtome (Leica, Germany). The slices were thaw mounted on gelatine-covered microscope slides, air dried, and stored at −20 °C until use (Fig. 1).

For RT-qPCR and HPLC studies, the remaining brains were cut into 1-mm-thick coronal slices containing structures of interest by using cutting edge. Then, ventral tegmental area (bregma, −5.80 to −6.30 mm) and prefrontal cortex (bregma, 3.70 to 1.70 mm) were punched out, by using a tissue puncher, from tissue slices containing the above structures, according to The Rat Brain in Stereotaxic Coordinates Atlas (Paxinos and Watson 1998). All tissue samples were frozen on dry ice in Eppendorf tubes and stored at −80 °C until use.

### miRNA Purification from Serum and Brain Tissue Samples

Total RNA including miRNA from serum and brain tissue samples was purified by using miRNeasy Serum/Plasma Kit (Qiagen, Germany) and miRNeasy Mini Kit (Qiagen, Germany), respectively. All purification procedures were performed according to attached protocols combined with the use of a QIAcube robotic workstation (Qiagen, Germany) for automated purification of total RNA including miRNA. Synthetic ath-miR-159a miRNA mimic (Life Technologies, CA, USA) was spiked into QIAzol Lysis Reagent to a final concentration of 4 fmol/ml in order to control quality of the RNA purification, complementary DNA (cDNA) synthesis, and PCR amplification in the qPCR experiment. Purity and concentration of purified RNA samples were measured with the use of NanoDrop spectrophotometer (Thermo Scientific, MA, USA). Samples with good quality were taken for further RT-qPCR analysis.

### Real-Time qPCR Analysis of miRNA Expression and Data Normalization

One microgram of total RNA from brain samples and 3 μl of total RNA from serum samples were reverse transcribed into cDNA by using the TaqMan® MicroRNA Reverse Transcription Kit (Life Technologies, CA, USA) and custom-made TaqMan® MicroRNA reverse transcription primer pool (Life Technologies, CA, USA) according to the procedure for multiplexing the RT step without preamplification while using TaqMan® MicroRNA Assays. Custom-made TaqMan® MicroRNA RT pool consisted of a mixture of 0.05× specific stem-looped RT primers for miR-18a-5p, miR-34a-5p, miR-135a-5p, miR-195-5p, miR-320-3p, miR-674-3p, miR-872-5p, ath-miR-159a-5p, miR-106b, and let-7i (Life Technologies, CA, USA) dissolved in 1× TE buffer (Sigma-Aldrich, Germany). This set of miRNAs was selected based on our previous observations and literature showing that miRNAs mentioned above may be associated with stress response and functioning of the nervous system. Then, 6 μl of cDNA from each sample was mixed with 50 μl of TaqMan® Universal PCR Master Mix, No Amperase® UNG (Life Technologies, CA, USA), and 44 μl of nuclease-free water (Sigma-Aldrich, Germany) to a final volume of 100 μl. Then, the cDNA mixture was loaded into the custom-made TaqMan® Low Density Cards (TLDA) (Life Technologies, CA, USA). RT-qPCR reaction was run on the QuantStudio™ 12K Flex system (Life Technologies, CA, USA) by using the default thermal cycling conditions and the automatic baseline and threshold values in accordance with the manufacturer’s protocol. Raw Ct values were analyzed by using qubasePLUS software (Biogazelle, Belgium). Ct values were normalized by using the delta–delta Ct method and converted to relative quantities for NormFinder and geNorm analysis. The relative quantities of target miRNAs were normalized by using qubasePLUS software to the geometric means of miR-106b and let-7i which showed stable expression and thus meet the criteria of reference targets.

### Bioinformatic Comparative Analysis of Prediction of SERT–miRNA Interaction

We used miRWalk2.0—online available software and database (http://zmf.umm.uni-heidelberg.de/apps/zmf/mirwalk2/index.html)—which shows miRNA binding sites within the complete sequence of rat genome (including 5′-UTR, 3′-UTR regions as well as coding sequences) and combines this information with a comparison analysis of binding sites predicted by other existing miRNA target prediction algorithms such as miRNAMap, RNAhybrid2.1, or TargetScan6.2. We classified particular miRNA as potential SERT mRNA regulator if any of the abovementioned miRNA target prediction algorithms (included in comparison analysis provided by miRWalk2.0) revealed miRNA–SERT interaction. The list of miRNAs potentially involved in SERT posttranscriptional regulation together with algorithms that documented this interaction is shown in Table 1.

**Table 1:** List of miRNAs of interest which showed potential interaction with SERT based on miRWalk2.0 comparison analysis of binding sites predicted by four different algorithms

Gene	miRNA	miRWalk	RNAhybrid	miRMap	TargetScan
slc6a4	miR-18a-5p		1		
slc6a4	miR-34a-5p	1	1		
slc6a4	miR-135a-5p	1	1	1	1
slc6a4	miR-195-5p	1	1	1	1
slc6a4	miR-320-3p	1	1		
slc6a4	miR-674-3p	1	1		
slc6a4	miR-872-5p		1		

1 means positive interaction revealed by a particular algorithm

### [^3^H]Paroxetine Binding to Serotonin Transporter and Analysis of Autoradiograms

Tissue sections were preincubated in 50 mM Tris–HCl buffer (pH 7.4) with 300 mM NaCl at room temperature for 15 min. Then, the brain slices were incubated for 90 min at room temperature in 50 mM Tris–HCl (pH 7.4) incubation buffer containing 300 mM NaCl and 1 nM [^3^H]paroxetine (specific activity 15.5 Ci/mmol). Non-specific binding was determined by using 10 μM fluoxetine. After incubation, tissue sections were washed 2 × 2 h in 50 mM Tris–HCl buffer (pH 7.4) with 300 mM NaCl at 4 °C and once in ice-cold distilled water. The sections were dried overnight under a stream of air. The labeled with [^3^H]paroxetine brain slices were placed against an imaging plate (Fujifilm, Japan) with autoradiographic microscales (GE Healthcare) for 7 days. After this time, plates were developed by using FujiFilm BAS-5000 Phosphoimager (FujiFilm, Japan). The obtained autoradiograms were analyzed and quantified by using ImageGauge software (Fujifilm, Japan). The specific binding of radioligand was calculated by subtracting non-specific binding images in adjacent brain slices from the total binding signal. The results are expressed in femtomole of bound radioligand per milligram of tissue (fmol/mg) in each examined structure.

### SERT mRNA In Situ Hybridization

Brain tissue coronal slices were fixed in 4 % formaldehyde for 10 min on ice, washed in ice-cold PBS buffer for 5 min, and then incubated for 10 min in an ice-cold acetic anhydride (0.25 %)—TEA (0.1 M) solution. Fixed brain slices were then dehydrated in ethanol and subjected to two 10-min incubations in chloroform. Next, slices were washed again in ethanol and air dried. A mixture of three commercially available oligonucleotides complementary to the rat SERT mRNA was used in the in situ hybridization assay. SERT oligonucleotide probe sequences were as follows:

5′GCATCTACCCACACCCCTGTCTCCAAGAGTTTCTGCCAGTTGGG3′; 5′ACGATGAGCACGAACCATTCCCTGCGCTTGGCCCAGATGTGA3′; 5′ATCCGCTCCTTAAGTGTCCCCGGAGTGCTGATCAGCCGATAAATG3′.

Oligonucleotide probes were labeled at the 3′ end with [^35^S]dADP (Hartmann Analytic, Germany) by using terminal transferase (Fermentas, Lithuania). The probes were suspended to a final concentration of 1 pmol per 200 μl of hybridization buffer containing the following components: 1× Denhardt’s solution (0.02 % polyvinylpyrrolidone/0.02 % Ficoll/0.02 % bovine serum albumin), 4× saline sodium citrate (SSC) (pH 7.0), 50 % (*v*/*v*) formamide, 10 % (*w*/*v*) dextran sulfate, yeast tRNA (0.25 mg/ml), salmon sperm ssDNA (0.2 mg/ml), and 10 mM dithiotreitol. Tissue slices were incubated with hybridization buffer containing radiolabeled probes for 18 h at 37 °C in humid conditions then washed four times (15 min each) in 2× SSC with 50 % formamide at 42 °C. The hybridized brain sections were rinsed with distilled water, dehydrated in ethanol, and air dried. Prepared tissue sections were placed into X-ray cassettes and exposed to film plates (Kodak) for 90 days at a temperature of −20 °C. The developed autoradiograms were analyzed, quantified, and normalized by using ImageGauge software (Fujifilm, Japan). Data are expressed as percent changes in mRNA signals compared with the control group (expressed as 100 %).

### Statistical Analysis

The effect of 2 weeks of CMS on sucrose consumption and time-dependent changes in serum levels of miRNAs were analyzed by using two-way ANOVA followed by Bonferroni’s post hoc test. *p* values from analysis of time-dependent changes in serum miRNAs were also corrected for multiple comparisons by using the correction method of Benjamini and Hochberg, controlling the false discovery rate (FDR) at an alpha level of 5 % [25]. One-way ANOVA analysis of variances followed by Tukey’s multiple comparison post hoc test was used to analyze changes in the expression level of miRNAs, changes in [^3^H]paroxetine binding to SERT, and changes in SERT mRNA expression in rat brain structures after 2 weeks of CMS. Statistical analysis of the data was performed by using GraphPad Prism5 software (GraphPad Software, Inc., La Jolla, CA, USA).

## Results

### The Effect of 2 Weeks of the CMS Procedure on Sucrose Consumption

**Fig. 2:**
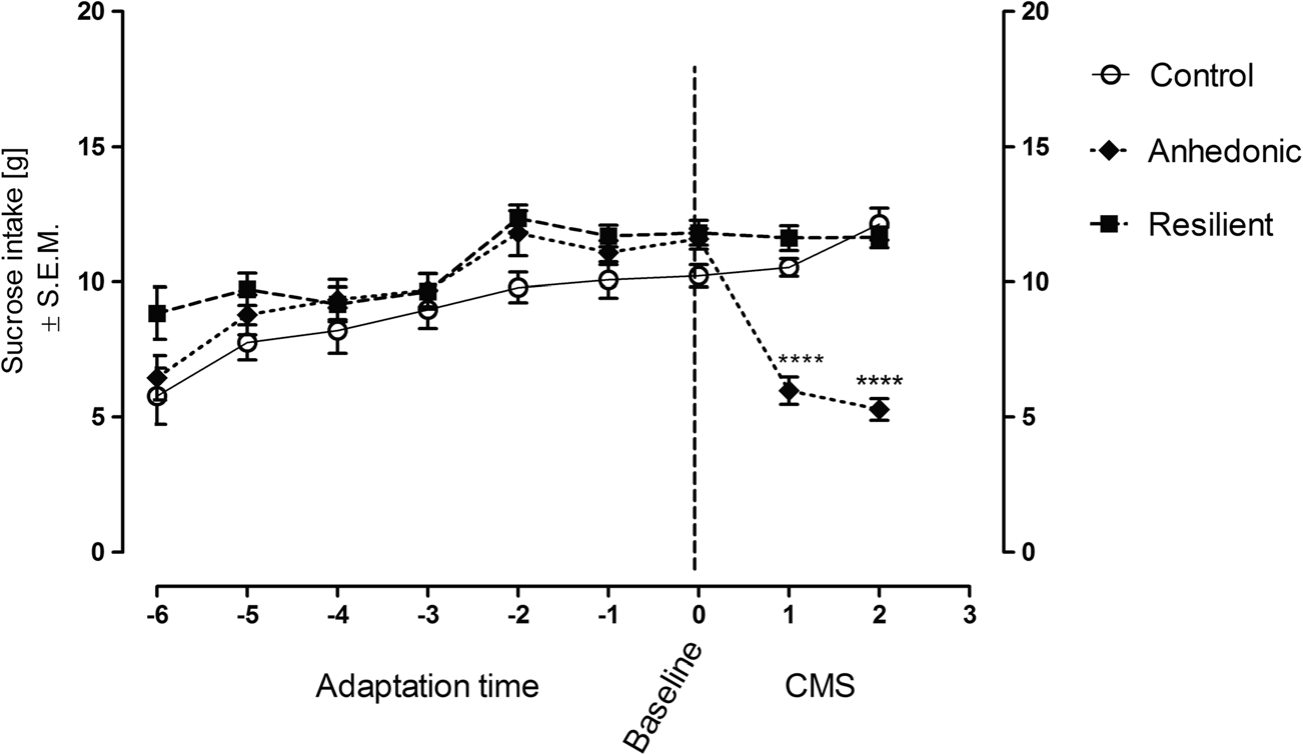
The effect of chronic unpredictable stress exposure on 1 % sucrose solution consumption [g] in control, anhedonic, and resilient animals. Time points from −6 to 0 represents consecutive sucrose testes of all animals during adaptation period. Adaptation represents time when animals were trained to drink 1 % sucrose solution. Time point 0 named “baseline” represents the last sucrose test before the start of the weekly stress regimen. After initiation of stress, anhedonic rats (after first week of stress) significantly decreased their sucrose consumption as compared to their basal sucrose consumption as well as to non-stressed controls and resilient animals which, in turn, despite being stressed did not change their sucrose consumption as compared to their baseline sucrose consumption and controls. Results were retrospectively analyzed by using repeated measures ANOVA followed by Bonferroni’s post hoc test. *n* = 14–16; ****p < 0.0001

After 2 weeks of the CMS procedure, repeated measures ANOVA revealed statistically significant effect of stress (*F*_2,129_ = 72.42; *p* < 0.0001), time (*F*_2,129_ = 14.57; *p* < 0.0001), and interaction stress × time (*F*_4,129_ = 26.57; *p* < 0.0001) on the consumption of a palatable sucrose solution by the rats (Fig. 2). Additionally, Tukey’s post hoc test showed that anhedonic group of rats after first week of CMS procedure significantly decreased sucrose consumption as compared to non-stressed and resilient groups of rats. This effect was maintained during the second week of the stress regimen. On the other hand, resilient group of animals despite being stressed did not change their sucrose consumption during CMS as compared to baseline level of sucrose consumption before the stress and as compared to control group of rats.

### The Effect of 2 Weeks of CMS on the Expression Levels of miR-18a-5p, miR-34a-5p, miR-135a-5p, miR-195-5p, miR-320-3p, miR-674-3p, and miR-872-5p in Mesocortical Pathway

**Fig. 3:**
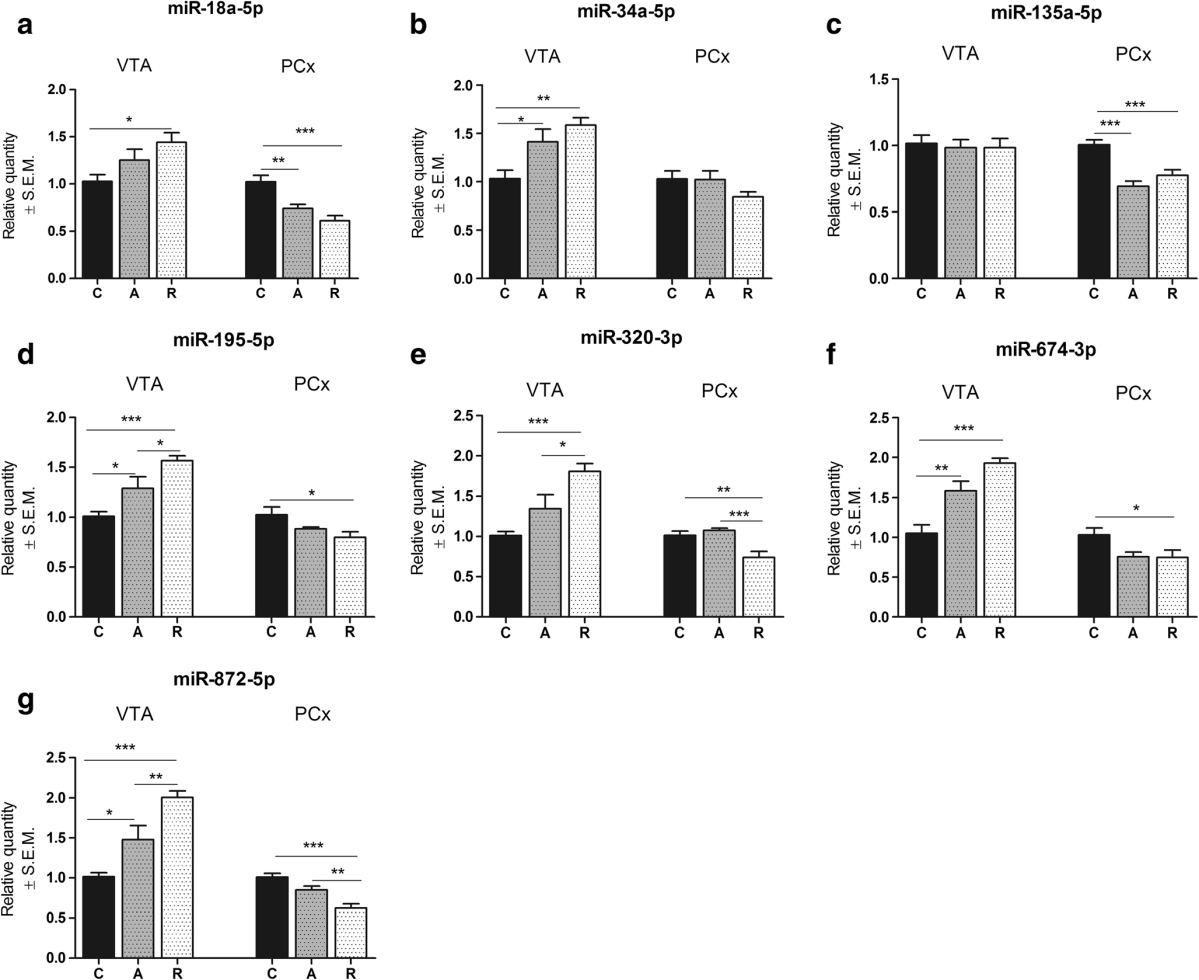
The influence of 2 weeks of CMS on the expression level of a set of seven miRNAs in mesocortical circuit of control (*C*), anhedonic (*A*), and resilient animals (*R*). *Left panel* of each graph represents particular expression in ventral tegmental area (*VTA*), and *right panel* represents results from miRNA expression in prefrontal cortex (*PCx*). miRNA expression represented as relative quantity with regard to control analyzed by one-way ANOVA followed by Tukey’s post hoc test. *n* = 10; *p < 0.05, **p < 0.01, ***p < 0.001

**Fig. 4:**
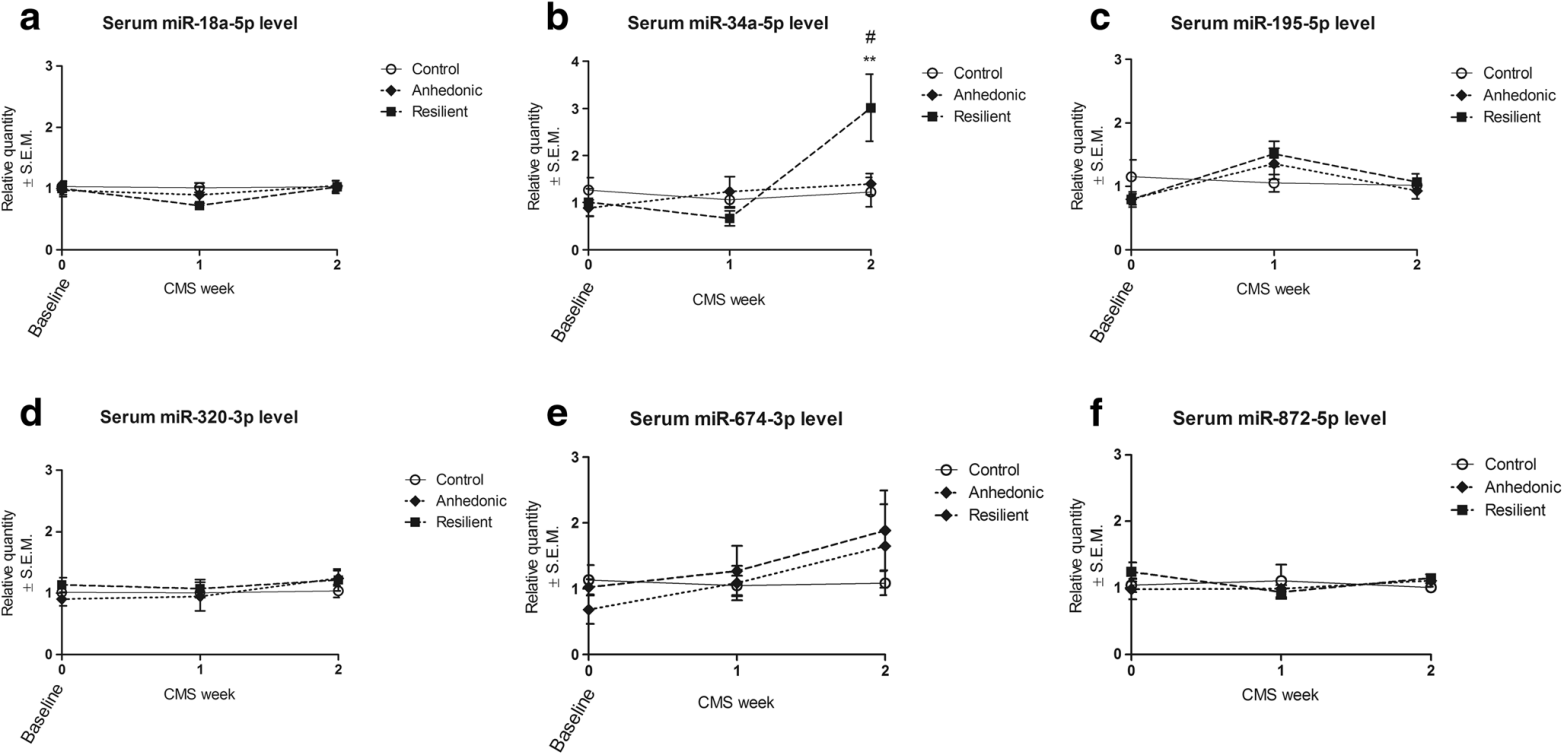
Time-dependent analysis of serum level of different miRNAs in control, anhedonic, and resilient animals during 2 weeks of CMS. Serum level of miR-135a-5p was undetectable. Baseline time point represents miRNA serum level in rats before the start of the stress regimen. Two-way ANONA followed by Bonferroni’s post hoc test revealed increased serum level of miR-34a-5p in resilient animals after 2 weeks of CMS as compared to control and anhedonic rats. *n* = 5–9; **p < 0.01 vs control, #*p* < 0.05 vs anhedonic

Our results clearly show that exposure to 2 weeks of CMS strongly affected miRNA expression in mesocortical pathway (Fig. 3). One-way ANOVA revealed increased expression levels of all miRNAs of interest (except miR-135a-5p, see Fig. 3c) after 2 weeks of CMS in VTA (Fig. 4a, b, d–g) of stressed animals as compared to non-stressed group of rats (miR-18a-5p *F*_2,29_ = 4.34, *p* < 0.05; miR-34a-5p *F*_2,29_ = 8.03, *p* < 0.01; miR-195-5p *F*_2,29_ = 12.88, *p* < 0.001; miR-320-3p *F*_2,29_ = 11.31, *p* < 0.001; miR-674-3p *F*_2,29_ = 19.99, *p* < 0.0001; miR-872-5p *F*_2,29_ = 18.18, *p* < 0.0001). This effect was even more profound among resilient group of animals as compared to control and anhedonic rats as showed by post hoc analysis (Fig. 3a, b, d, e, g). Moreover, statistical analysis showed that stress exposure for two consecutive weeks also decreased the expression levels of all examined miRNAs (except miR-34a-5p, see Fig. 3b) in rat PCx (Fig. 3a, c–f) as compared to control group of rats (miR-18a-5p *F*_2,29_ = 14.19, *p* < 0.0001; miR-135a-5p *F*_2,29_ = 16.95, *p* < 0.0001; miR-195-5p *F*_2,29_ = 4.13, *p* < 0.02; miR-320-3p *F*_2,29_ = 10.57, *p* < 0.001; miR-674-3p *F*_2,29_ = 4.03, *p* < 0.05; miR-872-5p *F*_2,29_ = 15.67, *p* < 0001). Again, post hoc analysis revealed that impact of stress on the expression level of different miRNAs was greater in resilient group of rats as compared to control and anhedonic animals (Fig. 3d–g).

### Dynamic Changes in the Serum Levels of miR-18a-5p, miR-34a-5p, miR-135a-5p, miR-195-5p, miR-320-3p, miR-674-3p, and miR-872-5p during 2 Weeks of the CMS Procedure

We analyzed time-dependent changes in serum levels of seven miRNAs which have a potential to interact with SERT (see Table 1) in order to find whether peripheral and dynamic changes in the levels of miRNAs mentioned above may be associated with behavioral state of the animals. miR-135a-5p was not detectable in rat serum. The rest of the six miRNAs were present in serum. At the “baseline” time point (i.e., before the start of the stress procedure), all animals exhibited similar levels of all assayed miRNAs (Fig. 4). Two-way ANOVA analysis of variances showed significant effect of time (*F*_2,54_ = 5.81; *p* < 0.01) and interaction stress × time (*F*_4,54_ = 3.86; *p* < 0.01) on the serum level of miR-34a-5p (Fig. 4b). Tukey’s post hoc analysis together with the correction of the *p* values for multiple comparisons showed that serum level of miR-34a-5p was significantly elevated after 2 weeks of CMS in resilient group of animals as compared to control and anhedonic rats (Fig. 4b).

### The Effect of Stress Exposure on the Expression Level of SERT Protein in Mesocortical Circuit and the Level of mRNA Encoding SERT in Dorsal Raphe Nucleus

**Fig. 5:**
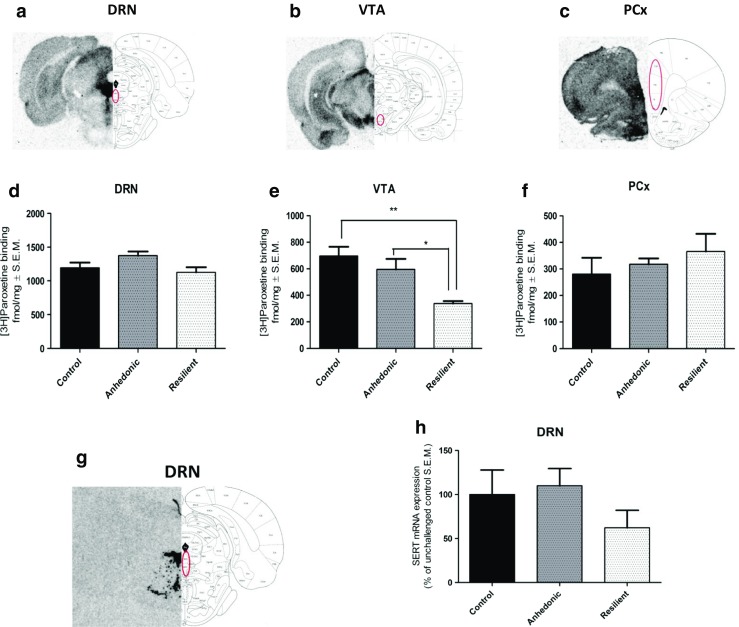
Representative autoradiorgams of specific [^3^H]paroxetine binding to SERT protein in DRN (**a**), VTA (**b**), and PCx (**c**) and SERT mRNA (**g**) in coronal sections of the rat brain. Brain structures were dissected according to Paxinos and Watson (1998). The effect of stress response on SERT level was investigated in DRN (**d**), VTA (**e**), and PCx (**f**). **h** SERT mRNA expression in DRN. One-way ANOVA analysis followed by Tukey’s post hoc test showed significant decrease in [^3^H]paroxetine binding to SERT protein in VTA of resilient animals as compared to control and anhedonic rats (**e**). *n* = 4–6; *p < 0.05 vs anhedonic, **p < 0.01 vs control

We examined the binding of [^3^H]paroxetine (selective serotonin reuptake inhibitor (SSRI)) to SERT in order to check whether global changes in expression levels of different miRNAs potentially targeting SERT may be associated with altered SERT protein level in vivo in mesocortical circuit. There was no difference in the SERT level in dorsal raphe nucleus (DRN) and PCx of animals after 2 weeks of CMS (Fig. 5d, f). Nevertheless, [^3^H]paroxetine binding to SERT protein showed decreased level of SERT in VTA (*F*_2,11_ = 9.15, *p* < 0.01) of resilient rats after 2 weeks of CMS as compared to control and anhedonic groups of animals (Fig. 5e).

In situ hybridization analysis showed no difference in the level of mRNA encoding SERT in DRN in animals after 2 weeks of CMS (Fig. 5h).

## Discussion

Coping strategies exhibited by an organism in the face of stress are essential to minimize its negative impact. Depression may result from insufficient adaptation of the brain to adverse environmental stimuli. miRNAs have a potential to regulate allostasis of the brain in the face of stress and may contribute to develop the depression through acting on major signaling pathways induced by stress in different brain circuits [6]. It is becoming clear that miRNAs are involved in the control and maintenance of normal physiological functioning of the central nervous system [3, 5]. Animal models of depression which can discriminate susceptible from resilient subjects are of prime importance in order to fully understand complex mechanisms underlying allostatic balance in the face of stress. Moreover, the examination of the differences between subjects susceptible and resilient to stress may give rise to the development of a new, promising treatment of depression, based on the restoration of resilient phenotypes among depressed individuals. In our present study, we used CMS—the aforementioned animal model of depression. In CMS, mild and chronic stressors are used in order to mimic natural psychosocial stress often disturbing people without being related to trauma. Moreover, importantly, the CMS model (with the exception of anhedonic rats) discriminates resilient animals which, despite being stressed, maintain normal behavior. Although CMS is an expensive, time-consuming animal model, it is well suited for experiments related to a natural and differential stress response.

RNA interference, initiated in mammals mainly by miRNA molecules, is considered to be a new and upstream biochemical level of posttranscriptional regulation of gene expression importantly involved in buffering changes in response to stress and thus allostatic balance. It is very likely that a natural variety in miRNA expression among different subjects may contribute to individual reaction to stress [26]. Defining miRNA expression pattern in different brain circuits, as well as in peripheral tissues, may lead to the molecular characterization of resilient and susceptible phenotypes. In the present study, we focused on a set of seven miRNAs which are potentially involved in stress response and pathophysiology of depression.

Uchida et al. have shown that stress-vulnerable F344 rats exhibited increased expression level of miR-18a-5p in the hypothalamic paraventricular nucleus as compared to Sprague–Dawley animals [27]. Moreover, they have also shown that miR-18a-5p regulated the expression of glucocorticoid receptor [27]. miR-34a-5p is important in regulating the behavioral and neurochemical response to acute stress [8] and modulates neurogenesis and synaptogenesis [28]. miR-135a-5p has been shown to mediate anxiety-like and depression-like behaviors as well as antidepressant response [13]. miR-195-5p regulates serotonergic signaling and BDNF level—two biochemical pathways strongly involved in pathophysiology of mental illness [29]. Decreased plasma level of miR-320-3p has been observed in depressed and drug-naïve patients [12]. miR-674-3p and miR-872-5p have been shown to be associated with traumatic stress response [30]. Moreover, our bioinformatic comparative analysis by using miRWalk2.0 [31] which is an online freely accessible software showed that all abovementioned miRNAs have a potential to regulate SERT expression (see Table 1). SERT is one of the main targets of commonly used antidepressant drugs. Our previous results have revealed that the action of miR-16 in the mesocortical circuit may be associated with resilient phenotype [20].

Mesocortical circuit consists of VTA—a midbrain structure, conserved among higher ordered vertebrates, which projects neuronal connections to PCx and limbic structures. Dysregulation of a balance of mesocortical pathway under stress is responsible for cognitive aspects of depression such as impaired executive functioning [22]. Aversive as well as reward-related stimuli can cause rapid excitement of VTA neurons [32] which, in turn, change the functioning of PCx. Moreover, it has been shown that dopaminergic mediation in VTA is associated with a consumption of a palatable solution and lesions to the VTA selectively disturbed overconsumption of highly preferred solutions by the rats [33]. Additionally, Martinez-Hernandez et al. have shown that lesions of VTA reduced preference for sucrose in CD-1 female mice [34].

Smalheiser and co-workers have shown that antidepressant-free suicidal patients exhibited widespread decrease of miRNA expression in PCx as compared to matched healthy controls [35]. Our results showed that anhedonic animals exhibited decreased expression of two miRNAs potentially involved in negative regulation of SERT (miR-18a-5p and miR-135a-5p) in PCx as compared to non-stressed animals. Moreover, for the first time, we revealed that resilient animals exhibited decreased expression levels of almost all examined (except miR-34a-5p) miRNAs in PCx as compared to the control group of rats. Although there was no statistically significant effect of stress on miR-34a-5p expression level in PCx, resilient animals exhibited trend to have decreased level of this miR. It is noteworthy that resilient animals exhibited higher degree of decrease in the expression level of miRNAs of interest than anhedonic animals as compared to the control group of rats. In case of miR-320-5p and miR-872-5p in PCx, we observed that resilient animals were even more reactive to stress than anhedonic-like rats that was reflected by statistically significant change in the expression of both mentioned above miRNAs in resilient animals as compared to anhedonic-like group of rats. Decreased miRNA expression in PCx may result from hypo-activation of this structure in animals and, as shown by Smalheiser et al., human experiencing prolonged stressful conditions. Nevertheless, hypo-activation of PCx under stress at the level of miRNA biogenesis seems not to be sufficient to induce depressive symptoms as we showed that it occurs both among susceptible and resilient animals. This phenomenon may be a natural reaction to stress observed in both groups of animals and human and may be caused by negative feedback from other structures functionally connected to PCx such as VTA which projects directly to PCx creating mesocortical circuit. In our study, we observed an interesting relationship: Decrease in miRNA expression in PCx of stressed animals was accompanied by an increase in miRNA expression in VTA; however, it should be noted that we observed a lack of effect of miR-135a-5p in VTA, and this observation needs further investigation. Again, resilient animals exhibited more profound increase in miRNA expression in VTA than anhedonic rats as compared to non-stressed control group of rats (see Fig. 3). Our observation shows that mesocortical circuit is involved in maintaining allostatic balance under stress at the level of miRNA expression and it is more prevalent and dynamic in resilient than anhedonic subjects. Thus, we can conclude that resilient animals exhibited more effective coping strategy in the face of stress which was associated with more increased response in miRNA expression in mesocortical system among resilient animals as compared to both control and susceptible rats.

Although brain studies are more invasive and difficult to perform than when using tests of peripheral tissues (such as blood), they are still the most relevant, in order to explore the molecular basis of mental illness. Nevertheless, research aimed at finding peripheral markers reflecting changes associated with mental illness are equally important. Recent advances have revealed that miRNAs may be good candidates to act as a peripheral marker of stress response [7, 13, 36]. Katsuura, for example, and co-workers have shown that naturalistic stress in healthy students caused significant elevation of miR-144/144* and miR-16 in blood [37]. Additionally, it has been observed that resilient animals in CMS model exhibit elevated and time-dependent level of miR-16 in serum [20]. Sun et al. have shown that expression levels of miR-34c-5p and 34b-5p were significantly higher in peripheral blood leukocytes of depressed patients than healthy controls. Moreover, they have shown that suicide idea patients exhibited lower levels of miR-34b-5p and miR-369-3p [38]. In our study, we observed that resilient animals had increased serum level of miR-34a-5p as compared to non-stressed and anhedonic rats. This observation suggests that altered serum miRNA level may be one of the markers reflecting efficient coping strategies, without necessarily serving as a factor for determining resilient phenotype because basal level of miR-34a-5p was similar among all animals (see Fig. 3b). It is noteworthy that miR-34a-5p, miR-34b-5p, and miR-34c-5p are members of the same miRNA family, possessing identical seed region and therefore similar potential targets. Among all miRNAs tested in our study, only serum level of miR-34a-5p was altered after 2 weeks of CMS. It supports the hypothesis that miRNAs belonging to miR-34-5p/449-5p family may have the potential to be a peripheral marker of stress response especially since miR-34a-5p has been considered as specific for brain and spinal cord tissue [39]. However, further studies are needed to define the exact role of miR-34-5p/449-5p family in stress-related pathologies because it has been shown that both resilient animals and depressed patients exhibit increased miR-34 levels.

Recent data suggest that miRNAs play an important role in cellular response to stress as well as antidepressant action by regulating the serotonergic system [40–42]. It is also confirmed that VTA receives rich serotonergic input from dorsal and medial raphe nuclei [43]. This suggests that serotonin signaling can coordinate VTA functioning which, in turn, may influence on behavior related to reward. Our bioinformatic analysis has shown that all miRNAs tested in our study have a potential to regulate SERT level. Thus, we focused on SERT changes in mesocortical circuit which may be associated with miRNA alterations. We observed that there was no difference in SERT level in all assayed structures between anhedonic and control animals in CMS model, which is consistent with Little’s research showing that there was no significant difference in [^125^I]RTI-55 binding to SERT in VTA between depressed subjects and controls [44]. However, we have revealed that resilient animals exhibited significantly decreased SERT protein level in VTA as compared to anhedonic and control rats (see Fig. 5) and this alteration accompanied increased expression in miRNAs which are involved in negative regulation of SERT. Additionally, in situ hybridization analysis showed no difference in SERT mRNA level in DRG in all tested animals which supports the hypothesis that SERT is engaged in response to stress and is regulated by miRNAs in VTA. This effect may reflect compensatory attempts taken by an organism in the face of a challenging condition. Unfortunately, due to technical limitations, we could not perform RT-qPCR analysis of changes in miRNA expression levels in DRG, and this needs further study.

Our findings show that mesocortical circuit is involved in stress response and this phenomenon is more efficient in resilient animals. This response is shown at the level of miRNA expression which, in turn, influenced downstream biochemical pathway associated with SERT expression. It also opens, together with recent advances, new investigative avenue in studies on the pathophysiology of mental illness at the level of miRNA regulation.
